# Adverse late health outcomes among children treated with 3D radiotherapy techniques: Study design of the Dutch pediatric 3D‐RT study

**DOI:** 10.1002/cnr2.1620

**Published:** 2023-01-30

**Authors:** Josien G. M. Beijer, Judith L. Kok, Geert O. Janssens, Nina Streefkerk, Andrica C. H. de Vries, Cleo Slagter, John H. Maduro, Petra S. Kroon, Martha A. Grootenhuis, Eline van Dulmen‐den Broeder, Jacqueline J. Loonen, Markus Wendling, Wim J. E. Tissing, Helena J. van der Pal, Marloes Louwerens, Arjan Bel, Jaap den Hartogh, Margriet van der Heiden‐van der Loo, Leontien C. M. Kremer, Jop C. Teepen, Cécile M. Ronckers

**Affiliations:** ^1^ Princess Máxima Center for Pediatric Oncology Utrecht The Netherlands; ^2^ Department of Radiation Oncology University Medical Center Utrecht Utrecht The Netherlands; ^3^ Department of Pediatric Oncology Erasmus Medical Center Rotterdam The Netherlands; ^4^ Department of Radiation Oncology Erasmus Medical Center Rotterdam The Netherlands; ^5^ Department of Radiation Oncology, University of Groningen University Medical Center Groningen Groningen The Netherlands; ^6^ Department of Pediatric Oncology/Hematology Amsterdam University Medical Center/Vrije Universiteit Amsterdam Amsterdam The Netherlands; ^7^ Department of Hematology Radboud University Medical Center Nijmegen The Netherlands; ^8^ Department of Radiation Oncology Radboud University Medical Center Nijmegen The Netherlands; ^9^ Department of Pediatric Oncology, Beatrix Children's Hospital University Medical Center Groningen Groningen The Netherlands; ^10^ Department of Internal Medicine Leiden University Medical Center Leiden The Netherlands; ^11^ Department of Radiation Oncology Amsterdam University Medical Center/University of Amsterdam Amsterdam The Netherlands; ^12^ Dutch Childhood Cancer Parent Organization Nieuwegein The Netherlands; ^13^ Department of Pediatrics, Emma Children's Hospital Amsterdam University Medical Center/University of Amsterdam Amsterdam The Netherlands; ^14^ University Medical Center Utrecht, Wilhelmina Children's Hospital Utrecht The Netherlands

**Keywords:** pediatric radiotherapy, childhood cancer survivors, side effects, study design, radiation dose effects

## Abstract

**Background:**

Adverse late health outcomes after multimodal treatment for pediatric cancer are diverse and of prime interest. Currently available evidence and survivorship care guidelines are largely based on studies addressing side‐effects of two dimensional planned radiotherapy.

**Aims:**

The Dutch pediatric 3D‐planned radiotherapy (3D‐RT) study aims to gain insight in the long‐term health outcomes among children who had radiotherapy in the 3D era. Here, we describe the study design, data‐collection methods, and baseline cohort characteristics.

**Methods and Results:**

The 3D‐RT study represents an expansion of the Dutch Childhood Cancer Survivor study (DCCSS) LATER cohort, including pediatric cancer patients diagnosed during 2000–2012, who survived at least 5 years after initial diagnosis and 2 years post external beam radiotherapy. Individual cancer treatment parameters were obtained from medical files. A national infrastructure for uniform collection and archival of digital radiotherapy files (Computed Tomography [CT]‐scans, delineations, plan, and dose files) was established. Health outcome information, including subsequent tumors, originated from medical records at the LATER outpatient clinics, and national registry‐linkage. With a median follow‐up of 10.9 (interquartile range [IQR]: 7.9–14.3) years after childhood cancer diagnosis, 711 eligible survivors were identified. The most common cancer types were Hodgkin lymphoma, medulloblastoma, and nephroblastoma. Most survivors received radiotherapy directed to the head/cranium only, the craniospinal axis, or the abdominopelvic region.

**Conclusion:**

The 3D‐RT study will provide knowledge on the risk of adverse late health outcomes and radiation‐associated dose‐effect relationships. This information is valuable to guide follow‐up care of childhood cancer survivors and to refine future treatment protocols.

## 1 INTRODUCTION

Treatment for childhood cancer has improved considerably over the last decades, which has led to a steady increase in the long‐term survival rate.^1^, ^2^ The present 5‐year survival rate after childhood cancer is approximately 80% in Europe.^1^, ^3^ Most childhood cancer patients receive multimodal treatment, with varying combinations of chemotherapy‐regimens, sometimes including hematopoietic stem cell transplantation (HSCT), radiotherapy, surgery and more recently, targeted therapy.^4^, ^5^, ^6^ Although treatment is necessary for cure, unfortunately, all modalities potentially affect normal tissues which can result in adverse late health outcomes.^7^, ^8^

To gain insight in the occurrence of and risk factors for these health outcomes, several childhood cancer survivors (CCS) cohorts in Europa, Northern‐America and Australia/New Zealand have been established.^7^, ^9^, ^10^, ^11^, ^12^ These studies have yielded a wealth of data on the health status, quality of life, and health care needs of the growing population of (young) adults with a history of pediatric cancer treatment, with implications for clinical care.^13^, ^14^, ^15^, ^16^ The majority of survivors included in these cohorts were diagnosed during 1940s–1990s when two dimensionally‐planned radiotherapy (2D‐RT), planning without Computed Tomography (CT) imaging, was the standard of care.^9^, ^10^ Since then, CT‐based three dimensionally‐planned radiation treatment (3D‐RT) and multi‐beam radiation delivery techniques have been implemented in pediatric oncology, sometimes referred to as ‘conformal radiotherapy’.^17^ Compared to 2D‐RT, the dose from 3D‐RT delivered to healthy tissues surrounding the tumor in the high‐dose region is generally lower at the cost of a larger area of healthy tissue receiving low‐dose irradiation.^18^, ^19^ While lower rates of harmful side effects can be expected due to reductions in high‐dose volumes^18^, ^19^ the long‐term effects of exposing more organs at risk (OARs) to the so‐called low‐dose bath remains unknown.^20^ As such, the validity of extrapolating currently available evidence on late effects generated from data on 2D‐RT treatments to patients treated in the 3D‐era remains uncertain. Although small studies on specific childhood cancer types have reported on side effects of 3D‐RT,^21^ large‐scale observational studies including follow‐up of patients beyond the 5‐year survival mark are rare to date.^22^

This study has expanded upon the resources of the Dutch Childhood Cancer Survivor Study (DCCSS) LATER Cohort of 5‐year CCS^10^, ^11^, ^23^ with the purpose of filling this evidence gap. In particular, the overall aim of the Dutch Pediatric 3D‐RT study is to evaluate adverse late health outcomes among children treated with radiotherapy in the era of CT‐based treatment planning, and to assess dose response relationships for selected health outcomes. In addition, our sub‐aims are to compare the frequency of late health outcomes in this cohort to children treated with 2D‐RT and to evaluate self‐reported health‐related quality of life. The current report describes the study design, data collection methods, and baseline characteristics of the 3D‐RT study.

## 2 METHODS

### 2.1 Study population/inclusion criteria

**FIGURE 1:**
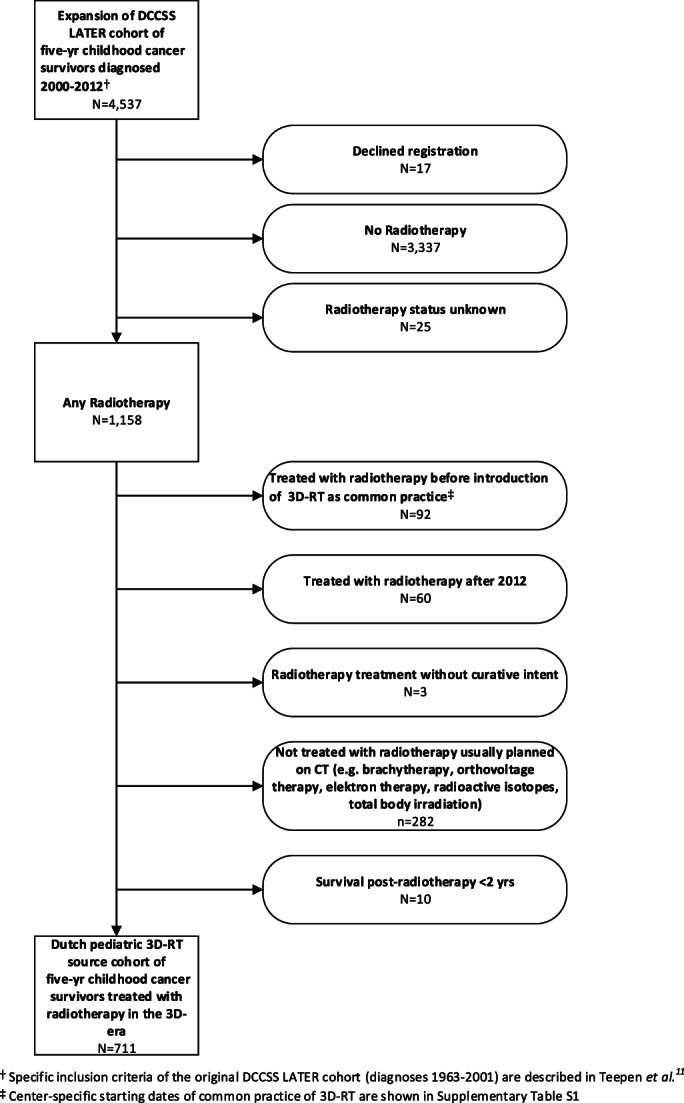
Flow diagram of the inclusion of childhood cancer survivors in the Dutch pediatric 3D‐RT study

Eligible survivors for the Dutch pediatric 3D‐RT study were identified from the DCCSS LATER cohort. The original DCCSS LATER cohort^11^, ^24^ included patients diagnosed in the period 1963–2001. Recently, we have expanded the cohort with patients diagnosed after this date. The base source of our study includes 4537 patients (Figure 1) diagnosed with a malignant neoplasm or selected other neoplasia^25^ before the age of 18 years, during 2000–2012, in a Dutch pediatric oncology center, and who survived at least 5 years after diagnosis. The 3D‐RT study includes a sub‐cohort with all patients who received external beam radiotherapy (EBRT) with curative intent planned in the 3D‐era, defined as the period since introduction of CT‐planning in the respective academic radiotherapy center, excluding total body irradiation (TBI), for a primary tumor and/or a recurrence. One Dutch pediatric oncology center was excluded (Table S1), because virtually all pediatric cancer patients treated with radiotherapy in this center received TBI as conditioning regimen for HSCT. HSCT was the local focus of this center; TBI treatment was an exclusion criterion for our study.

During the data collection phase, we identified potentially eligible 5‐year survivors of childhood cancer who received radiotherapy during the calendar period since introduction of CT‐based treatment planning. The calendar year of introduction of CT‐based treatment planning in routine care for children, and thus the start year of inclusion for this cohort study varied by center (2000, 2001 or 2002) in the six included centers (Table S1). We aimed to collect all digital radiotherapy files available in the centers. The detailed information on who did and who not did undergo 3D radiotherapy is currently being evaluated by use of the dose files (number and direction of beams) in combination with the information derived from the letter of the pediatric radiation oncologist based on radiotherapy technique used. Owing to the focus of the study on long‐term health outcomes rather than acute side effects, we excluded patients who passed away within 2 years after their first episode of radiotherapy.

In summary, the 3D‐RT study source cohort includes 5‐year survivors of childhood cancer in the Netherlands who fulfill the eligibility criteria for the DCSS LATER cohort, who received a form of EBRT with curative intent for a primary tumor or recurrence in the 3D‐era, defined as the period since introduction of CT‐planning in the respective academic radiotherapy department.

### 2.2 Data collection

#### 2.2.1 Cancer diagnosis and basic treatment details

Details on initial demographic details, prior cancer diagnosis, and treatment were abstracted by trained data managers in the pediatric oncology centers for all >10 000 5‐year survivors in the DCCSS LATER cohort. Basic treatment details includes details on surgery, radiotherapy (e.g., prescribed dose, radiotherapy field/location, boosts, start and end dates, and number of fractions, chemotherapy (drug names, start and end dates, and, for selected agents administered dose), HSCT, and other supportive medication for primary cancer and any recurrences. During additional 3D‐RT study data collection, missing basic radiotherapy details were complemented. In the future, treatment data for subsequent malignancies occurring during follow‐up will be additionally collected and added to the database.

#### 2.2.2 Radiotherapy files data collection

In the 3D‐RT study, a national infrastructure to facilitate organ‐specific dose assessment was created for storing digital radiotherapy files from all involved institutes, which is hosted at the department of Radiation Oncology of the University Medical Center Utrecht (UMCU). Digital radiotherapy files are collected in six radiotherapy departments and subsequently sent to the UMCU. This data collection is currently ongoing. Each patient‐specific radiotherapy file consists of three types of data: CT images, target and OAR delineation files, and dose files (including plan parameter files). CT images are used by the radiation oncologist to delineate the target volume and the OARs surrounding the tumor, which enables the design of a multi‐beam treatment plan sparing the OARs where feasible. The delineations are then saved in the delineation file and the dose information is saved in the dose file. Specific radiation parameters, such as the number of beams, beam angles, beam energy, and usage of multi leaf collimator rotation, are saved in the plan file.

The digital radiotherapy files cover radiotherapy for primary tumor and/or recurrences and, where applicable and feasible, for subsequent malignancies. Prior to centralization, the files are pseudonymized by replacing identifying information (e.g., medical record number, name, full date of birth, full treatment date, name of the attending physician or treating radiation oncologist/radiotherapy technician) by an identification number that corresponds with the number assigned during baseline data collection using software, such as Conquest,^26^ or custom‐made solutions. Hereafter the pseudonymized files are transferred to the centralized platform in UMCU. VolumeTool (version 1.29), a software program developed in the UMCU,^27^ is used to combine information from all three files. Dose volume histograms and corresponding volume information are abstracted from the files for the target volume and relevant OARs. As the prescribed dose is already available, this information will be presented here. The specific organ dose has to be calculated from the radiotherapy files data collection and potential additional delineation. The organ dose will be used in the following late effects analyses.

The completeness and quality of existing delineations differs depending on treatment period, protocol and guidelines used by centers. To avoid inter‐and intra‐observer variability of delineations performed by clinical experts in the existing delineation file, we will replace them by use of an automatic segmentation tool to accurately delineate all organs at risk according to current clinical practice. By using this automatic segmentation tool, we can systematically compare contoured organs at risk and thereby reduce inter‐ and intra‐observer variability. This automatic segmentation tool is used to delineate all organs at risk, except for small organs, for example, the pituitary gland. These organs at risks will be delineated manually by our research assistant with comprehensive training in delineations (LCW). An experienced radiation technologist (JLK) and an experienced pediatric radiation oncologist (GOJ) are consulted to check all delineations of the organs at risk used for research purposes. The detailed information on technical aspects of treatment planning is currently being analyzed, based on the dose files (e.g., number and direction of beams), and, for treatment delivery, also taking into account the information derived from the letter of the pediatric radiation oncologist that describes the radiotherapy technique use.

The retrieval and centralization of digital radiotherapy files is in various stages of completion in participating centers. Selection of relevant organs for dose assessment has been completed for organs in the head/neck area and, accordingly, delineations, are in progress.

#### 2.2.3 Adverse late health outcomes

Eligible adverse late health outcomes are based on a set of clinically relevant health outcomes for survivorship research proposed by Streefkerk et al.^28^ supplemented with specific radiotherapy‐specific side effects, such as alopecia and breast atrophy (see Table S2).^29^, ^30^, ^31^, ^32^, ^33^ Health outcomes will be ascertained using multiple sources. As a first step, medical information was abstracted from medical records at the LATER outpatient clinics, in particular the letter of the late effects outpatient physician that provides detailed summaries of the medical history. The reported outcomes were verified based on more elaborate information from the attending specialist. Moreover, data was collected on surgery involving (sub‐) total organ removals, as well as organ transplantations during the follow‐up period of the study, owing to the risk‐altering effects of such interventions for certain health outcomes. All clinically relevant medical outcome data, including date of occurrence, were registered on a data collection form and subsequently were entered in Castor EDC (Castor Electronic Data Capture, v2021.3.1.).

Furthermore, information about benign and malignant subsequent tumors will be based on record linkage with the nationwide network and registry of histo‐ and cytopathology in the Netherlands (PALGA)^34^ and the Netherlands Cancer Registry (NKR),^35^ for each eligible survivor. These procedures are in progress.

### 2.3 Ethics

The Princess Máxima Center institutional review board evaluated and approved the 3D‐RT study protocol, and the Medical Research Ethics Committee (MREC) Utrecht (protocol number: 20‐138/C) qualified the protocol as exempt from formal review according to the ‘Medical Research Involving Human Subjects Act’ (WMO), based on the retrospective nature and type of data collection. Similarly, the pre‐existing DCCSS LATER registry was declared exempt from review by the institutional review boards of the participating centers.

## 3 RESULTS

The national 3D‐RT study includes 711 5‐years CCS (male (n= 395; 56%)) treated with EBRT with curative intent (Figure 1). The median age at first radiotherapy was 10.6 years (interquartile range [IQR]: 5.7–14.5). The median age at follow‐up was 20.5 years (IQR: 16.7–24.9), and the median follow‐up time was 10.9 years (IQR: 7.9–14.3) after childhood cancer diagnosis. The median follow‐up time after first radiotherapy was 10.1 years (IQR: 7.4–13.2). The most common cancer diagnoses were Hodgkin lymphoma (*n* = 120; 17%), medulloblastoma (*n* = 90; 13%) and nephroblastoma (*n* = 62; 9%) (Tables 1, 2).

**TABLE 1:** Demographic and clinical characteristics in a nationwide 3D‐RT cohort of 711 5‐year survivors of childhood cancer treated with 3D planned radiotherapy during 2000–2012 in the Netherlands

Characteristics	Median (IQR)	N	%
Sex			
Female		316	44.4
Male		395	55.6
Age at first radiotherapy, years^a^	10.6 (5.7–14.5)		
0–4		119	16.7
5–9		185	26.0
10–14		228	32.1
15+		179	25.2
Age at last follow‐up, years	20.5 (16.7–24.9)		
0–9		20	2.8
10–19		286	40.2
20–29		360	50.6
30+		45	6.3
First course of 3D‐RT^a^			
Primary tumor		600	84.4
Recurrent tumor^b^		111	15.6
Diagnosis of childhood cancer^25^			
Leukemias, myeloproliferative diseases and myelodysplastic diseases		28	4.0
Lymphomas and reticuloendothelial neoplasms		138	19.4
CNS and miscellaneous intracranial and intraspinal neoplasms		251	35.3
Neuroblastoma and other peripheral nervous cell tumors		26	3.7
Retinoblastoma		3	0.4
Renal tumors		68	9.6
Hepatic tumors		0	0
Bone tumors		44	6.2
Soft tissue and other extraosseous sarcomas		89	12.5
Germ cell tumors, trophoblastic tumors, and neoplasms of gonads^c^		38	5.3
Other specified and unspecified malignant neoplasms^d^		25	3.5
Unknown		1	0.1
Calendar year of first course of 3D‐RT^a^			
2000–2003		139	19.5
2004–2007		241	33.9
2008+		331	46.6
Treatment group			
Radiotherapy (RT) only		12	1.7
RT + chemotherapy (C)		160	22.5
RT + surgery (S)		107	15.5
RT + C + S		341	48.0
RT + C + HSCT		23	3.2
RT + C + S + HSCT		60	8.4
Radiotherapy; Status other therapy unknown		8	1.1
Vital status at end of follow‐up^e^			
Alive		652	91.7
Deceased		59	8.3

Abbreviations: 3D‐RT Study, 3D‐planned Radiotherapy Study; IQR, interquartile range; CNS, central nervous system; RT, radiotherapy; C, chemotherapy; S, surgery; HSCT, hematopoietic stem cell transplantation.

^a^ First course of 3D‐CRT is the first radiotherapy following the start of 3D‐CRT in the specific center (Table S1).

^b^ These survivors entered the cohort based on EBRT for a recurrence.

^c^ 36 germ cell tumors originated from a CNS location.

^d^ Includes ICCC‐group “Other malignant epithelial neoplasms and malignant melanomas” (*n* = 24) and ICCC‐group “Other and unspecified malignant neoplasms” (*n* = 1).

^e^ : based on information in the medical file at the LATER outpatient clinics.

**TABLE 2:** Distribution of cohort members according to type of childhood cancer or exposed body parts and prescribed dose category (3D‐RT Study 2000–2012)

Type of childhood cancer	Number of survivors	Dose (Gy)^a^					
		<20	20–29	30–39	40–49	≥50	Unknown
Leukemias, myeloproliferative diseases and myelodysplastic diseases	28	13	13	‐	‐	‐	2
Lymphoid leukemias	21	12	8	‐	‐	‐	1
Other	7	1	5	‐	‐	‐	1
Lymphomas and reticuloendothelial neoplasms	138	7	101	23	6	‐	1
Hodgkin lymphomas	120	1	98	20	‐	‐	1
Other	18	6	3	3	6	‐	‐
CNS and miscellaneous intracranial and intraspinal neoplasms	251	2	5	9	10	225	‐
Ependymomas	53	‐	1	2	‐	50	‐
Choroid plexus tumor	2	‐	‐	‐	‐	2	‐
Astrocytoma	61	1	‐	1	‐	59	‐
Medulloblastoma	90	‐	4	4	8	74	‐
Other	45	1	‐	2	2	40	‐
Neuroblastoma and other peripheral nervous cell tumors	26	3	17	3	1	2	‐
Retinoblastoma	3	‐	‐	‐	2	1	‐
Renal tumors	68	47	19	2	‐	‐	‐
Nephroblastoma	62	45	15	2	‐	‐	‐
Other	6	2	4	‐	‐	‐	‐
Bone tumors	44	3	3	1	6	31	‐
Osteosarcomas	6	‐	2	1	‐	3	‐
Ewing tumor or Askin's tumor	38	3	1	‐	6	28	‐
Soft tissue and other extraosseous sarcomas	89	‐	3	3	28	54	1
Rhabdomyosarcomas	53	‐	1	3	21	28	‐
Ewing tumor or Askin's tumor	12	‐	2	‐	6	4	‐
Other	24	‐	‐	‐	1	22	1
Germ cell tumors, trophoblastic tumors, and neoplasms of gonads^b^	38	‐	5	‐	17	14	2
Other specified and unspecified malignant neoplasms^c^	25	‐	‐	1	‐	24	‐
Unknown	1	‐	1	‐	‐	‐	‐

Exposed body part	Number of survivors	Dose (Gy)^a^					
Head only	240	12	18	4	28	175	3
Spine only	25	2	5	2	1	15	‐
Cranio‐spinal	126	7	4	7	15	93	‐
Neck	26	‐	8	3	2	12	1
Thorax	71	9	34	9	9	10	‐
Abdominopelvic	109	44	31	4	9	21	‐
Extremities	25	‐	‐	2	3	18	2
Multiple locations^d^	87	1	64	12	3	7	‐
Unknown location	2	‐	2	‐	‐	‐	‐
Total	711	75	166	43	70	351	6

Abbreviations: 3D‐RT Study, 3D‐planned Radiotherapy Study; Gy: Gray; CNS: Central Nervous System.

^a^ First course of 3D‐RT is the first radiotherapy following the start of 3D‐RT in the specific center (Table S1). If primary and boost are administrated, both were summed.

^b^ 36 germ cell tumors originated from a CNS location.

^c^ Includes ICCC‐group “Other malignant epithelial neoplasms and malignant melanomas” (*n* = 24) and ICCC‐group “Other and unspecified malignant neoplasms” (*n* = 1).

^d^ Exposed body region of initial radiotherapy treatment to the primary cancer. In case the radiotherapy to the primary cancer comprised exposure to multiple body regions (e.g., neck and/or thorax and/or abdominopelvic region in Hodgkin lymphoma survivors), it was coded as “multiple locations.”

In 600 survivors (84%), EBRT was administered during the first line treatment. Of them, 64 (9%) survivors also received radiotherapy for one or more recurrence(s). The remaining 111 survivors (16%) entered the cohort after receiving EBRT for a recurrence. During the study period, cumulative treatment exposures included chemotherapy for 82% of survivors while 71% had a surgical procedure and 12% had HSCT (Table 1).

The median total prescribed dose to the primary tumor (dose to primary tumor plus boost dose, if applicable) was 45.6 Gy (IQR: 21.6–54.0 Gy). About one third of the survivors received radiotherapy to the head/cranium only (*n* = 240, 34%), 126 survivors to the craniospinal axis (18%) and 109 survivors received abdominopelvic radiotherapy (15%).

Table 2 represents the prescribed dose stratified by body regions and childhood cancer type, respectively. In all, 351 (49%), 166 (23%) and 75 (11%) of the survivors received a dose exceeding 50 Gy, 20–30 Gy, or less than 20 Gy, respectively. When stratified by childhood cancer type, doses exceeding 50 Gy were mainly observed among survivors of central nervous system tumors and sarcomas, while doses up to 30 Gy were mainly seen in survivors of lymphomas and renal tumors. Table 3 represents an overview of the radiated body part per childhood cancer type.

**TABLE 3:** Distribution of cohort members by type of childhood cancer to exposed body part (3D‐RT Study 2000–2012)

Type of childhood cancer	Exposed body part^a^								
	Head only	Spine only	Cranio‐spinal	Neck	Thorax	Abdominopelvic	Extremities	Multiple locations^d^	Unknown location
Leukemias, myeloproliferative diseases and myelodysplastic diseases	17	2	6	‐	1	1	1	‐	‐
Lymphomas and reticuloendothelial neoplasms	6	‐	3	12	36	4	3	72	2
CNS and miscellaneous intracranial and intraspinal neoplasms	139	7	105	‐	‐	‐	‐	‐	‐
Neuroblastoma and other peripheral nervous cell tumors	1	5	‐	‐	4	13	‐	3	‐
Retinoblastoma	3	‐	‐	‐	‐	‐	‐	‐	‐
Renal tumors	‐	‐	‐	‐	8	58	‐	2	‐
Bone tumors	2	9	‐	‐	14	13	6	‐	‐
Soft tissue and other extraosseous sarcomas	33	2	‐	8	8	19	15	4	‐
Germ cell tumors, trophoblastic tumors, and neoplasms of gonads^b^	24	‐	12	‐	‐	1	‐	1	‐
Other specified and unspecified malignant neoplasms^c^	15	‐	‐	6	‐	‐	‐	4	‐
Unknown	‐	‐	‐	‐	‐	‐	‐	1	‐
Total^d^	240	25	126	26	71	109	25	87	2

Abbreviations: 3D‐RT study, 3D‐planned radiotherapy study; CNS, central nervous system.

a First course of 3D‐RT is the first radiotherapy following the start of 3D‐RT in the specific center (Table S1). If primary and boost are administrated, both were summed.

^b^ 36 germ cell tumors originated from a CNS location.

^c^ Includes ICCC‐group “Other malignant epithelial neoplasms and malignant melanomas” and ICCC‐group “Other and unspecified malignant neoplasms.”

^d^ Exposed body region of initial radiotherapy treatment to the primary cancer. In case the radiotherapy to the primary cancer comprised exposure to multiple body regions (e.g., neck and/or thorax and/or abdominopelvic region in Hodgkin lymphoma survivors), it was coded as “multiple locations.”

Information on prescribed doses on an individual‐patient level has been collected for the cohort and is presented in this report. This characteristic enables comparisons across cohorts of childhood cancer survivors who had radiotherapy as part of their treatment regimen. Moreover, we are in the process of estimating the first series of absorbed doses as well as the volumetric aspects of radiation exposure, to specific organs and tissues in the head‐ and neck area. The resulting future library of radiation exposure to specific organs and tissues will be used in planned and future new late effects analyses.

## 4 DISCUSSION

In this paper, we described the study design, data collection, and baseline characteristics of the Dutch pediatric 3D‐RT study. The aim of our work is to refine insight in the risks of specific late adverse health outcomes occurring in children treated with radiotherapy in the era of CT‐based radiotherapy techniques and to investigate dose–response relationships for late health effects. In order to achieve this, we designed a multi‐center resource to collect and harmonize digital radiotherapy files that allows for future organ‐specific radiation dose (re)calculation among CCSs treated in the 3D era. With the future results of this project, we aim to provide empiric knowledge to enhance both treatment planning guidance in pediatrics, as well as surveillance guidelines for the follow‐up of childhood cancer survivors treated in the 3D era.

Although 3D‐RT represents the standard of care for pediatric cancer, the current evidence on health outcomes among CCSs is based on historic cohorts of mainly 2D‐RT treated patients, with a different radiation dose distribution. Because of this difference in dose distribution, insight in late adverse events following 3D‐RT is important. Our recent systematic literature review of observational studies on adverse health outcomes after 3D‐RT included 13 studies, most of which describe small patient cohorts (range of *N*: 5–246) with a median follow‐up of 8.8 years,^22^ and showed that evidence on this topic is scarce. Most available studies to date reported on selected late health outcomes. Our study is designed to address the full spectrum of adverse late somatic health outcomes, and, as such, can address both the total burden of disease as well as potential patterns of related late health effects.^28^ Also, our cohort is about three times larger (*N* > 700) than the largest study included in the review (*N* = 246). Finally, the studies that were eligible for the systematic review typically report on the prescribed dose rather than including organ doses and dose/volume aspects of radiotherapy exposure.

At present time, the only comprehensive prospective assessment of health outcomes after 3D‐RT, is the Münster‐based “Register of Treatment‐Associated Late Effects After Radiotherapy of Malignant Diseases in Childhood and Adolescence” (RiSK).^36^ The RiSK collaborative group collects data based on voluntary reporting from radiotherapy departments across Germany to the study center, concerning radiation dose parameters for clinically delineated OARs and health outcomes registered during regular follow‐up visits at the radiotherapy department in the initial post‐treatment period.^37^, ^38^

The study cohort described here will be suitable to describe the overall burden of disease and the incidence of specific health outcomes among children treated with radiotherapy in the 3D‐RT era in the Netherlands, but will also serve as source cohort to select subgroups of patients of specific interest, based on childhood cancer type, type of treatment planning or radiation delivery technique (e.g., intensity‐modulated radiotherapy), or area of radiation exposure. In addition, a series of nested case control studies will evaluate the effect of received dose on the surrounding OARs on occurrence of health outcomes. Of note, full characterization of the treatment technique and radiation beams among the children treated in the 3D‐RT era to date awaits completion of the full evaluation of the digital radiotherapy files, because relevant technical details are not always described in medical files or physician letters. Therefore, the source cohort of 711 survivors represents a group of children who received EBRT in the era when it was possible to use CT‐planning, although not all will necessarily have received 3D‐RT. For example, for patients with nephroblastoma^39^ neuroblastoma or lymphoma,^40^, ^41^ classic AP‐PA beam set‐ups have remained in use, in compliance with international trial protocols in effect during the calendar period covered by our cohort study, although the treatment planning did involve a CT‐scan in the study period. Similar considerations hold for craniospinal axis irradiation techniques.

A few limitations bear mentioning, although all are related to intrinsic treatment and patient characteristics that determine eligibility for research studies aimed at delivering novel empirical evidence required to tailor clinical practice.^42^, ^43^ Foremost, the median follow‐up period is comparatively short, in view of the long latency for many known side effects of radiotherapy in children, including subsequent malignancies and normal tissue effects. This fact cannot be changed anno 2022, since 3D‐RT was introduced very gradually since the late 1990s. Also, some of the required digital files were documented and stored based on long outdated treatment planning software. For most eligible patients, the radiation physicist managed to convert (parts of) the relevant information to a common DICOM standard. For files considered irretrievable, other sources of treatment details can be used for crude assessment of dose to certain OARs,^44^, ^45^, ^46^ so that valuable observation time for these patients treated early, can be included for selected analyses.

The study derives its main strength from the combination of multiple unique features. Radiotherapy files form the basis to derive valid and precise estimates of doses‐volume parameters to relevant organs and we have the opportunity to ascertain health outcomes from LATER outpatient clinics operating under a coordinated common umbrella and harmonized care plans, supplemented with record linkage to national disease registries. Outcome assessment is based on a rigorous data manual, in line with definitions proposed for harmonization of survivorship research^28^ and according to standards advocated for radiotherapy outcomes research.^47^ Once established, the study platform serves as basis to supplement the data with self‐reported information that cannot easily be captured from medical files or disease registries, pending funding. This may include data from the KLIK platform (Dutch: Kwaliteit van Leven In Kaart [Quality of Life in Clinical Practice]), a platform for Health Related Quality of Life questionnaires which can be used in daily clinical practice to facilitate the communication between physician and patient during consultation, empowers patients, and promotes shared decision making^48^, ^49^ and/or questionnaire surveys to the entire eligible cohort or those visiting the LATER outpatient clinic, as shown in previous research from the DCCSS LATER Consortium.^28^, ^50^ As such, this resource is available for times to come to address new research questions as they emerge in clinical practice. Also, as with all survivorship efforts, it is important to keep following this cohort to allow for evaluation of potential health problems among 3D‐RT survivors as they enter later phases of life.

Overall, this effort embraces and builds upon valuable collaborative work in clinical research^51^, ^52^ and late effects care and research^11^, ^50^ in academic centers in the past decades in the Netherlands coordinated by the former Dutch Childhood Oncology Group (DCOG) and continued in the Princess Máxima Center for Pediatric Oncology. It is important for the multidisciplinary treatment teams caring for survivors of childhood cancers and for survivors themselves to have accurate information about the potential late effects following radiation treatment. This information can update surveillance guidelines for the follow‐up of CCSs, such as those published by the International Guideline Harmonization Group (IGHG) or ‘Pediatric Normal Tissue Effects in the Clinic’ research collaboration.^53^, ^54^, ^55^, ^56^

In conclusion, this study described the study design, baseline characteristics and data collection of the 3D‐RT study, in which extensive data on radiotherapy and health outcomes are being collected for a nationally cohort of 711 5‐year CCS treated in the era of pediatric 3D radiotherapy techniques in the Netherlands. This study can provide new insights into risks of adverse late health outcomes in CCS treated with 3D‐RT and specific relations with radiation dose and volume.

## AUTHOR CONTRIBUTIONS

**Josien G. M. Hazewinkel‐Beijer:** Data curation (lead); formal analysis (lead); investigation (lead); methodology (equal); supervision (equal); visualization (lead); writing – original draft (equal); writing – review and editing (lead). **Judith L. Kok:** Data curation (supporting); investigation (supporting); methodology (equal); supervision (equal); writing – original draft (equal); writing – review and editing (lead). **Geert O. Janssens:** Data curation (supporting); funding acquisition (supporting); methodology (equal); resources (equal); writing – review and editing (supporting). **Nina Streefkerk:** Methodology (equal); resources (equal); writing – review and editing (supporting). **Andrica C. H. de Vries:** Resources (equal); writing – review and editing (supporting). **Cleo Slagter:** Resources (equal); writing – review and editing (supporting). **John H. Maduro:** Conceptualization (supporting); resources (equal); writing – review and editing (supporting). **Petra S. Kroon:** Resources (equal); writing – review and editing (supporting). **Martha A. Grootenhuis:** Conceptualization (supporting); writing – review and editing (supporting). **Eline van Dulmen‐den Broeder:** Resources (equal); writing – review and editing (supporting). **Jacqueline J. Loonen:** Resources (equal); writing – review and editing (supporting). **Markus Wendling:** Resources (equal); writing – review and editing (supporting). **Wim J. E. Tissing:** Resources (equal); writing – review and editing (supporting). **Helena J. van der Pal:** Methodology (equal); resources (equal); writing – review and editing (supporting). **Marloes Louwerens:** Resources (equal); writing – review and editing (supporting). **Arjan Bel:** Resources (equal); writing – review and editing (supporting). **Jaap den Hartogh:** Conceptualization (supporting); writing – review and editing (supporting). **Margriet van der Heiden‐van der Loo:** Resources (equal); writing – review and editing (supporting). **Leontien C. M. Kremer:** Conceptualization (supporting); funding acquisition (supporting); investigation (supporting); methodology (equal); project administration (equal); supervision (equal); writing – original draft (equal); writing – review and editing (supporting). **Jop C. Teepen:** Data curation (supporting); formal analysis (supporting); investigation (supporting); methodology (equal); project administration (equal); supervision (equal); visualization (supporting); writing – original draft (equal); writing – review and editing (lead). **Cécile M. Ronckers:** Conceptualization (lead); data curation (supporting); funding acquisition (lead); investigation (supporting); methodology (equal); project administration (equal); supervision (equal); writing – original draft (equal); writing – review and editing (lead).

## CONFLICT OF INTEREST

C. Ronckers, Ph.D., reported a personal grant from the Dutch Cancer Society (grant no: UVA2012‐5517). J. Beijer reported a grant from the Dutch Cancer Society (grant no: UVA2015‐7655). The other authors reported no conflicts of interest and did not receive funding.

## ETHICS STATEMENT

The Princess Máxima Center institutional review board evaluated and approved the 3D‐RT study protocol, and the Medical Research Ethics Committee (MREC) Utrecht (protocol number: 20‐138/C) qualified the protocol as exempt from formal review according to the ‘Medical Research Involving Human Subjects Act’ (WMO), based on the retrospective nature and type of data collection. Similarly, the pre‐existing DCCSS LATER registry was declared exempt from review by the institutional review boards of the participating centers.

## Supporting information


**TABLE S1**: Year of introduction of CT‐based treatment planning in routine‐care for children at the radiotherapy department.

**TABLE S2**: Definitions of potentially radiotherapy‐associated late health outcomes among childhood cancer survivors—Supplement to the standardized set of late health outcomes for survivorship research proposed by Streefkerk et al.^28^

## Data Availability

The data that support the findings of this study are stored in the Princess Máxima Center. Restrictions apply to the availability of these data, owing to the DCCSS LATER privacy protection regulations, which were used under license for this study. Data is not publicity available and is only available from the authors with permission of Princess Máxima Center.
