# Supplementary material for: Adverse late health outcomes among children treated with 3D radiotherapy techniques: Study design of the Dutch pediatric 3D‐RT study
Source: Cancer Rep (Hoboken). 2023 Jan 30;6(2):e1620. doi: 10.1002/cnr2.1620 (PMC9939987; doi:10.1002/cnr2.1620)
Supplement: Supplementary file 1 — TABLE S1: Year of introduction of CT‐based treatment planning in routine‐care for children at the radiotherapy department. [file CNR2-6-e1620-s001.docx]

**Supplementary Table S1: year of introduction of CT-based treatment planning in routine-care for children at the radiotherapy department**

| **Academic pediatric oncology center^†^** | **Year** |
| --- | --- |
| Amsterdam university medical center  Location Academic Medical Center | 2000 |
| Amsterdam university medical center  Location Vrije Universiteit Medical Center | 2000 |
| Radboud University Medical Center | 2002 |
| University Medical Center Groningen | 2000 |
| University Medical Center Utrecht | 2001 |
| Erasmus University Medical Center | 2000 |

†: Leiden University Medical Center was excluded because of local focus on total body

irradiation as conditioning regimen for stem cell transplantation.
