# Supplementary material for: Adverse late health outcomes among children treated with 3D radiotherapy techniques: Study design of the Dutch pediatric 3D‐RT study
Source: Cancer Rep (Hoboken). 2023 Jan 30;6(2):e1620. doi: 10.1002/cnr2.1620 (PMC9939987; doi:10.1002/cnr2.1620)
Supplement: Supplementary file 2 — TABLE S2: Definitions of potentially radiotherapy‐associated late health outcomes among childhood cancer survivors—Supplement to the standardized set of late health outcomes for survivorship research proposed by Streefkerk et al.28 [file CNR2-6-e1620-s002.docx]

| **Supplementary Table S2: Definitions of potentially radiotherapy-associated late health outcomes among childhood cancer survivors - Supplement to the standardized set of late health outcomes for survivorship research proposed by Streefkerk et al. ^[28]^**^†^ | | |
| --- | --- | --- |
|  | **Late health outcome** | **Definition of clinical relevance** |
| **Eye disorders** | Loss of vision | Loss of vision of at least one eye |
|  | Retinopathy | Disease involving the retina, requiring treatment, with at least one of the following criteria:  1. decreases of visual acuity  2. limiting daily life |
|  | Dry eye | Dryness of the cornea and conjunctiva, requiring artificial tears |
|  | Other eye disorders | Other eye-related disorder (excluding nystagmus, strabismus, amblyopia, correction eye position, eyelid correction, double vision) requiring treatment |
| **Ear conditions** | Other ear related conditions | Other ear related disorder requiring treatment or hearing aid |
| **Vascular conditions** | Other vascular conditions | Other vascular conditions with clinical symptoms, confirmed by a clinical evaluation with at least one of the following criteria:  1. requiring medication or  2. requiring surgical intervention |
| **Hepatobiliary conditions** | Other hepatobiliary conditions | Other hepatobiliary conditions with clinical symptoms, confirmed by a clinical evaluation with at least one of the following criteria:  1. requiring medication or  2. requiring surgical intervention |
| **Musculoskeletal conditions** | Fibrosis | Fibrosis is a fibrotic degeneration of the connective tissue, with restriction of locomotor movement diagnosed by a physician |
|  | Slipped capital femoral epiphysis | Capital femoral epiphysis displaces posteriorly on the femoral neck at the level of the physis diagnosed by a physician |
|  | Pathologic fracture | Fracture after minimal or no trauma in a weakened bone requiring surgery |
|  | Unequal limb length | Unequal limb length characterized by a discrepancy between the lengths of the lower or upper extremities, with at least one of the following criteria: 1. Discrepancy is 2 centimeters or larger 2. Indications of a shoe lift 3. Limitations in daily life |
| **Neoplasms** | Benign neoplasms | Benign neoplasm of any kind |
| **Skin tissue conditions** | Alopecia | Alopecia is a condition characterized by permanent loss of hair, compared to normal for a given individual at a given age and body location, in any degree persisting for 12 months after radiotherapy completion |
| **Fertility conditions** | Reduced fertility/subfertile | Condition characterized by reduced fertility with prolonged time of unwanted non-conception, with at least one of the following criteria:  1. not able to conceive after 12 months of unprotected intercourse  2. reduced ovarian reserve or impaired spermatogenesis (confirmed by laboratory testing)  3. fertility-enhancing treatment or assisted human reproduction (such as intracytoplasmic sperm injection, Intrauterine insemination, In vitro fertilization, testicular sperm extraction, egg cell or sperm donor, or surrogate mother) |
| **Pregnancy conditions** | Fetal death | Fetal loss at any gestational age |
|  | Fetal growth retardation | Fetal growth retardation resulting in the inability of the fetus to achieve its potential weight of at least 10% percentile of weight for gestational age |
|  | Premature delivery | Premature delivery with a viable infant at 28 to 34 weeks of gestation |
|  | Postpartum Hemorrhage | Postpartum hemorrhage is loss of 500 ml blood or more from the genital tract within 24 hours after birth of a baby |
| **Other conditions** | Breast atrophy | Condition characterized by underdevelopment of one of the breast including tanner stage (if available) diagnosed by a physician |
|  | Bilateral breast hypoplasia | Condition characterized by no/minimal development of both breasts including tanner stage (if available) diagnosed by a physician |
|  | Facial deformity | Facial asymmetry and hypoplasia (including ocular hypertelorism, ocular hypotelorism or deformity of the ear) diagnosed by a physician |
|  | Chronic pain | Chronic pain is a sensation of marked discomfort, with at least one of the following criteria:  1. requiring medical treatment i.e. with medication, surgical intervention, nerve stimulation or nerve blocks 2. limiting instrumental daily life |
|  | Hepatectomie | Status after hepatectomy |
|  | Hypoplasia | Asymmetry or decrease of tissue (muscle, bone or soft tissue) diagnosed by a physician |
| †: The radiotherapy-associated late health outcomes presented in this table were included as outcomes in addition to the set of outcomes for childhood cancer survivors defined by Streefkerk et al.^[28]^ . | | |
